# Prevalence and impact of Rotavirus A and C in suckling piglets from Spanish farms: an epidemiological study

**DOI:** 10.1186/s40813-025-00468-z

**Published:** 2025-10-30

**Authors:** Marcial Marcos-Cienfuegos, Francisco Javier Martínez-Lobo, M. Teresa Tejedor, Jaime Castillo-Pérez, Cinta Prieto

**Affiliations:** 1 MSD Animal Health, Salamanca, Spain; 2 Animal Science Department, School of Agrifood and Forestry Engineering and Veterinary Medicine, University of Lleida, Lleida, Spain; 3 Dpto. Anatomía, Embriología y Genética Animal, CIBER CV, Facultad de Veterinaria, Universidad de Zaragoza, Zaragoza, Spain; 4 Animal Health Department, Faculty of Veterinary Sciences, Universidad Complutense de Madrid, Madrid, Spain

**Keywords:** Porcine rotavirus, Prevalence, Neonatal diarrhea, Risk factors

## Abstract

**Background:**

Rotaviruses (RVs) are a leading cause of viral acute gastroenteritis in mammals, including pigs. Infection with porcine RVs can result in a range of clinical outcomes, from asymptomatic cases to severe acute disease. The prevalence of RVs is high in major pork-producing countries but varies by region, age group, and overall animal health. Several studies have assessed Rotavirus A (RVA) and Rotavirus C (RVC) prevalence in suckling piglets in Spain and other regions; however, systematic sampling studies remain limited. This study aimed to determine the true prevalence of RVA and RVC in suckling piglets across Spanish regions and to identify potential risk factors associated with infection.

**Results:**

A total of 563 fecal samples were collected from 106 farms, 84.5% from piglets with diarrhea and 15.5% from healthy piglets from farms without neonatal enteric disorders. RT-qPCR analysis revealed that RVA is a widespread pathogen in suckling piglets, with 43.7% of the samples and 74.5% of the farms testing positive. Conversely, RVC was detected in 25.4% of samples and 44.3% of the farms. The prevalence of RVA was higher in diarrheic piglets than in non-diarrheic ones (46.6% vs. 27.6%). Similarly, RVC prevalence was markedly higher in diarrheic compared to non-diarrheic piglets (29.2% vs. 4.6%). While RVA was detected throughout the lactation period, RVC was more frequently identified during the first week of life. For both viruses, higher viral load and proportion of positive animals were associated with enteric disorders during lactation, while RVC infection specifically correlated with increased mortality. Co-infections of RVA and RVC were relatively rare, suggesting that the presence of one virus may reduce the likelihood of detecting the other. Several risk factors were associated with rotavirus infection, including farm production type, farm size, and the duration of downtime in farrowing units.

**Conclusions:**

RVA and RVC are highly prevalent among suckling piglets, with a clear association between infection and diarrhea, particularly when viral loads are high. Farm production type, farm size, and management practices strongly influenced infection risk. These findings provide valuable epidemiological insights into RV infection in piglets and support the development of improved prevention and control strategies.

**Supplementary Information:**

The online version contains supplementary material available at 10.1186/s40813-025-00468-z.

## Background

Rotaviruses (RVs) are segmented double-stranded RNA viruses with 11 segments encoding six structural and five or six nonstructural proteins involved in infection and assembly [1]. Its triple-layered structure includes proteins VP2, VP6, VP7 and VP4, with VP4 and VP7 determining host range [2, 3]. Rotaviruses are classified into a total of nine species, named RVA to RVJ [4, 5].RVs are a major cause of acute viral gastroenteritis in mammalian and avian hosts, as these viruses cause villous atrophy, activation of the enteric nervous system, ischemia of the intestinal villi and an enterotoxigenic effect caused by NSP4 [2, 6]. Infection with porcine RVs could lead to different clinical outcomes, from an asymptomatic infection to the appearance of acute and severe clinical signs. Variations in disease manifestation are influenced not only by viral characteristics—such as strain-dependent virulence—but also by host-associated factors, including age, immunological status and general herd health [7]. At the cellular level, host susceptibility is further shaped by the viral preference for mature intestinal epithelial cells particularly in the jejunum and ileum. Among the molecular determinants of viral attachment, histo-blood group antigens (HBGAs) have been identified as critical glycan ligands mediating RV-host interactions in a genotype- and species-specific manner [8]. In swine, phenotypic variation in HBGA expression—driven by the porcine ABO system—modulates the efficiency of viral binding and internalization. Functional studies using porcine ileal enteroids have demonstrated that these glycan phenotypes markedly influence the replication dynamics of both RVA and RVC strains [8, 9], highlighting HBGA expression as a key host genetic factor governing RV infection outcomes *in vivo.*

Furthermore, management and production system variables play a significant role in shaping both the incidence and severity of disease outcomes. In particular, the management of the animals, the design of the facilities, and the health programs implemented, among other variables, have been described as risk factors or determinants of disease in RV enteric infections in pigs [7]. Regardless of these factors, the disease usually presents profuse watery diarrhea, lethargy, vomiting and anorexia, all accompanied by rapid wasting. Morbidity can reach 100%, but despite the clinical picture, lethality is rarely high (less than 20% of affected animals) [7].

The prevalence of RV is generally high among pork producing countries but varies by region, age group, and animal health status. In particular, several studies indicate that RVA is particularly prevalent in lactating and nursery pig, with some regions, such as North America, reporting infection rates as high as 84% [10]. In Asia, the prevalence of RVA ranges from 14 to 20% in piglets with diarrhea, while prevalence in non-diarrheic samples shows a significant variability, from 0% in Thailand to 25% in Vietnam. In Europe, the reported prevalence also exhibits a high degree of variability, with prevalences ranging from 6.5% in Ireland to 51.2% in Germany in samples originating primarily from diarrheic pigs [11, 12]. Within European countries, several studies have been carried out in Spain from 2012 onwards, and they indicate that the prevalence of RVA seems to be high in this country [13, 14], with percentages reaching up to 51.6% in diarrheic piglets [15].

On the other hand, the prevalence of RVC is particularly high in North America, reaching 59.5% in newborn piglets [16]. In contrast, in Asia and Europe, the prevalence is lower and more stable, ranging from 31 to 33% among diarrheic piglets. Remarkably, RVC is seldom identified in samples from non-diarrheic piglets. Thus, the virus was not detected in a study conducted in Spain [17] and was detected in only 4.4% of the samples in Ireland. However, a notable exception was observed in a study conducted in Spain, where 36.4% of control samples yielded positive results [15].

It is noteworthy that most published studies have analyzed samples submitted to diagnostic laboratories for the purpose of determining the etiology of enteric conditions. Consequently, these samples do not constitute an adequate representation of the population in study and do not include a control group, which limits their ability to provide comprehensive insights in the epidemiology of the RVs. Additionally, factors influencing the presentation and severity of RV diarrhea on pig farms are not often sufficiently addressed. A comparison of the prevalence of RVA and RVC infections with epidemiological data and actual farm management could offer valuable insights into the epidemiology of these viruses which could help to implement more effective prophylactic measures. Consequently, the objectives of this study were to determine the prevalence of RVA and RVC infection in suckling piglets on farms with and without a significant incidence of neonatal diarrhea from different Spanish regions, selected to adequately represent the Spanish swine population, and to identify possible risk factors for both viral infections.

## Methods

### Experimental design

A total of 106 sow farms distributed throughout Spain were included in this study. The number of farms sampled in each region was determined based on the regional inventory of breeding sows, as reported by the Ministry of Agriculture, Fisheries, and Food in its inventory of December 2022 [18] (Additional file 1). A total of 563 samples were received from these farms, with 84.5% representing diarrheal stools and the remainder 15.5% normal stools without diarrhea from farms without neonatal enteric disorders reported.

On each farm, the sampling protocol consisted in collecting 5 individual fecal samples from 5 different piglets, each from a different litter, during the lactation period. Clean gloves were used to collect each sample to avoid cross-contamination. Whenever possible, the samples were collected from piglets that had not been treated with any antimicrobials.

Samples were shipped immediately after collection under refrigeration to the Animal Health Department at the Faculty of Veterinary Sciences, Complutense University of Madrid, where they were labeled and stored at -80 °C until analysis. In cases where immediate shipment was not possible for logistical reasons, samples were frozen at -20 °C on the farm before being transported to the laboratory under refrigerated conditions.

### Epidemiological survey

To define farms with a high incidence of neonatal diarrhea for the purposes of this study, we conducted a questionnaire among participating farms. One survey item specifically addressed the prevalence of neonatal diarrhea and read as follows: “Is neonatal diarrhea a problem on your farm? Please answer Yes if more than 10–15% of litters are affected by diarrhea during the lactation period.” We established this cutoff based on the epidemiological situation in Spanish farms. Farms whose respondents reported that > 10–15% of litters experienced diarrhea during lactation were classified as having a high incidence of neonatal diarrhea.

To assess the different risk factors that may influence the prevalence of RVA and RVC infection, an epidemiological questionnaire was completed by the farmer, with the assistance of a veterinarian, immediately after the clinical samples were shipped. The questionnaire was designed and reviewed by the authors based on known or published risk factors to RV infection and other enteric viral infections in pigs [19] (Additional file 2).

### Sample processing

All samples underwent identical preparation prior to nucleic acid extraction. First, the fecal sample was weighed using a precision balance to determine its mass. Then, Phosphate Buffer Saline (PBS) (137 mM NaCl, 2.7 mM KCl, 10 mM Na₂HPO₄, and 1.8 mM KH₂PO₄, pH = 7.4) was added to each tube to create a 1:5 dilution (weight: volume). The mixture was then vortexed vigorously for at least 30 s or until completely homogenized. Finally, a 1 mL aliquot of the homogenized solution was removed and frozen at -80 °C until use.

### Nucleic acids extraction and detection of the presence of RVA and RVC in the samples by RT-qPCR

Extraction and purification of nucleic acids from fecal samples was carried out using the complex workflow protocol of the commercial MagMAX CORE Nucleic Acid Purification Kit (Life Technologies, Thermo Fisher Scientific Inc., Waltham, MA, USA) using a semi-automatic system (KingFisherTM, Life Technologies, Thermo Fisher Scientific Inc., Waltham, MA, USA).

RT-qPCR was performed using the commercial kits INgene q Rotavirus A and INgene q Rotavirus C (Gold Standard Diagnostics™, Madrid), which have good sensitivity and specificity values for the detection of RVA and RVC in clinical samples [20]. All reactions were performed on a 7500 Fast Real-Time PCR System, laptop, QST thermocycler (Applied Biosystems™, Thermo Fisher Scientific Inc., Waltham, MA, USA).

### Statistical analysis

To determine whether piglet age was a risk factor for RV infection, the samples were categorized into three groups: Group 1 included all samples from piglets 0 to 7 days old, Group 2 included all samples from piglets 8 to 15 days old and Group 3 included samples from piglets 16 days old or older.

Statistical analyses were conducted using IBM SPSS version 26 software (IBM Corp., Armonk, NY, USA). Prevalence of RVs has been assessed as a binary variable (presence /absence), measured as both percentage of positive farms and percentage of positive samples per farm. Comparison of percentages between diarrhea and control groups was performed using Pearson’s chi-square test or, alternatively, Fisher’s exact test, when appropriate. On the other hand, prevalence was also considered as a continuous variable, when percentage of positive animals per farm was measured. Both percentage of positive animals per fam and Ct values were submitted to the Shapiro -Wilk test for assessed normality. Comparison between two or more study groups for these continuous variables were performed using Analysis of Variance (ANOVA) for normally distributed data and non-parametric test (Mann-Whitney or Kruskal- Wallis test ) for non-normally distributed data. Multiple comparisons were adjusted using Bonferroni correction. To examine co-infections with RVA and RVC, the phi coefficient was calculated to assess the association between two binary variables (positive/negative). This coefficient quantifies the direction and strength of the relationship, with values ranging from − 1 to + 1. A value of + 1 indicates perfect agreement, while − 1 indicates complete disagreement. Associations were interpreted as strongly negative (-1 to -0.7), moderately negative (-0.7 to -0.3), or weak/not significant (-0.3 to + 0.3). Results were considered as statistically significant when p value was less than 0.05.

## Results

### Intra- and inter-farm prevalence of RVA and RVC

**Fig. 1:**
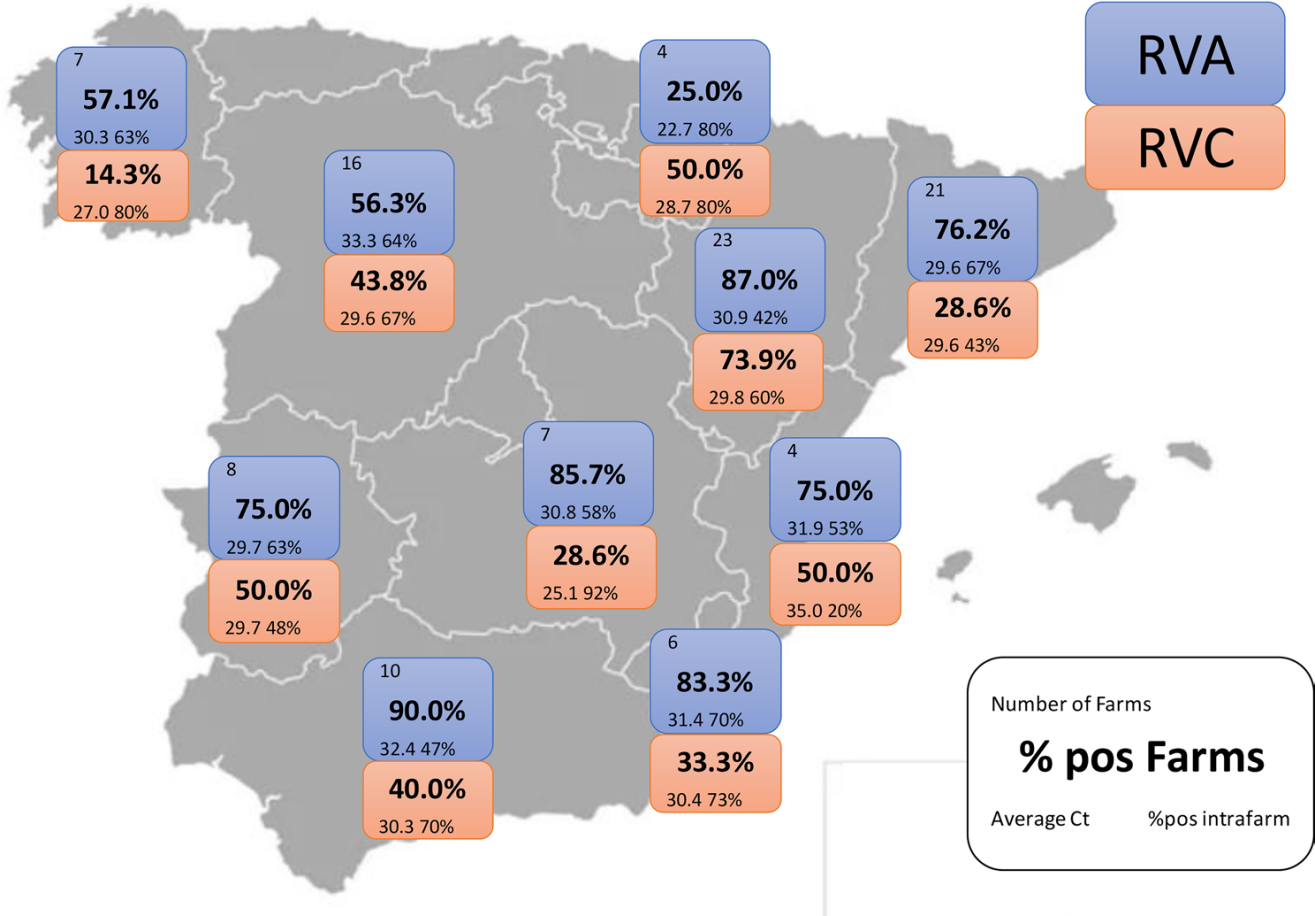
Geographic distribution of sampled herds and RV prevalence by region. The number of herds sampled, herd prevalence by region (Autonomous Community), mean detection frequency per herd (%) and mean Ct value are indicated for each region. Regions with no data represent areas where no samples were collected due to very low swine density

RVA was detected in 79 out of the 106 farms studied (giving an on-farm prevalence of 74.5%). When the on-farm prevalence was studied by region, the percentage of positive farms exceeded 56.3% in all the regions studied with the sole exception of Navarre, in northern Spain, where the prevalence was 25% (Fig. 1).

When the prevalence of RVA in individual animals was studied, 246 out of 563 samples analyzed were positive, giving a prevalence of 43.7% in animals. However, the percentage of positive samples varied widely between farms and across regions, ranging from 42% in Aragon to 80% in Navarre. The Ct values across all samples varied from 13.4 to 38.0. Nonetheless, the mean Ct values were relatively consistent among regions, ranging from 29.6 in Catalonia to 33.3 in Castile and Leon. The only exception was the sole positive farm in Navarre, where the mean Ct value was 22.7.

On the other hand, RVC was detected in 44.3% of the farms. Its prevalence across regions was consistently lower than that of RVA, remaining below 50%, except in Aragon, where it reached 73.9%. Ct values ranged from 14.5 to 38.0, with mean values varying notably by region, from 25.1 in Castilla-La Mancha to 35.0 in the Valencian Community (Fig. 1). Within-farm positivity also showed wide variability, ranging from 20% to 92%. At the national level, 25.4% of the individual samples tested positive.

### Frequency of detection of RV in fecal samples from animals with or without diarrhea

**Fig. 2:**
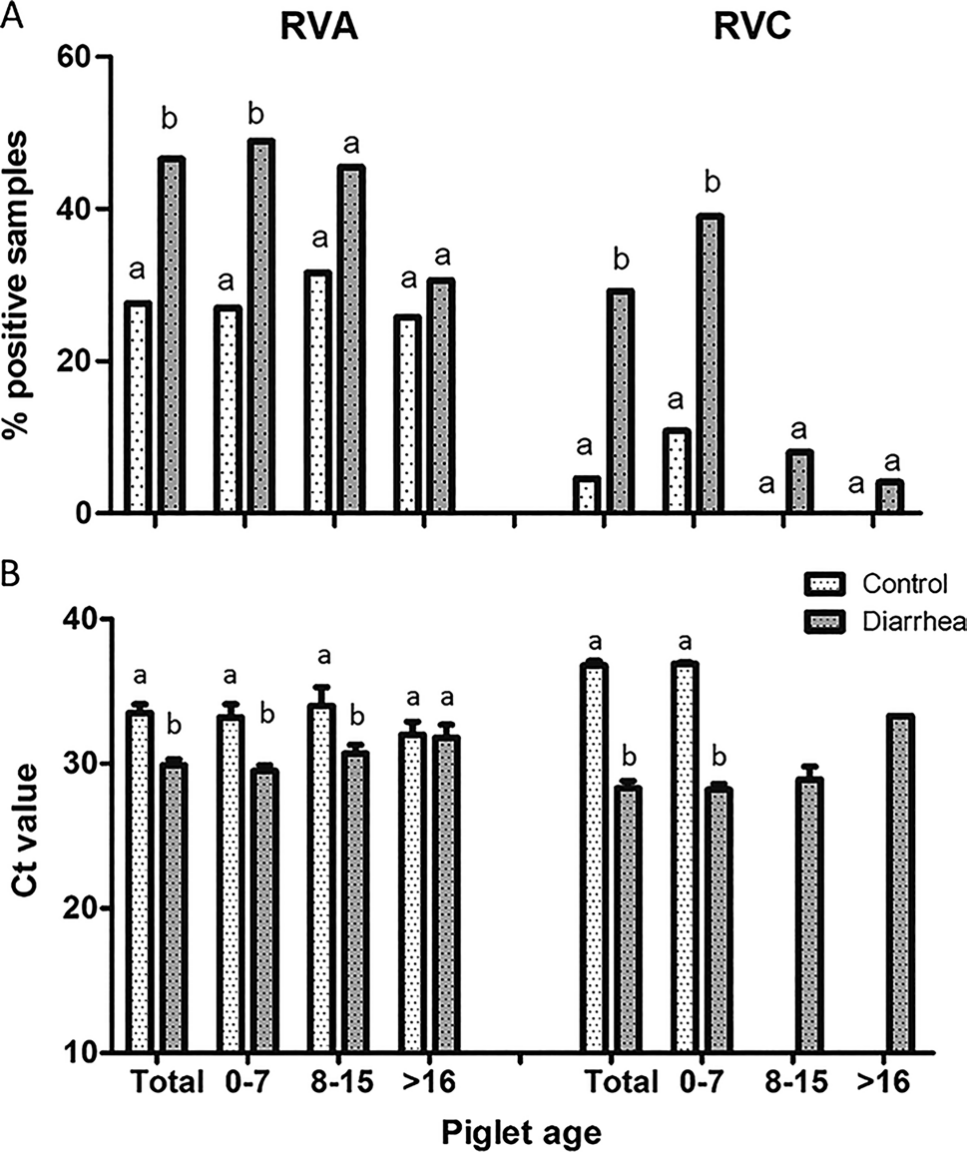
**A**) Percentage of fecal samples positive for RVA and for RVC from animals with and without diarrhea. **B**) Mean Ct values, and standard deviation, of RVA and RVC in fecal samples from animals with and without diarrhea. Mean values from all samples and distribution by piglet age are represented. *Different superscripts indicate statistically significant differences. (Comparison of percentages between diarrhea and control groups was performed using Pearson’s chi-square test)*

The frequency of detection of RVA was significantly different between animals with diarrheic feces and control animals with normal feces. Thus, 46.6% of the samples tested positive in the diarrhea group compared to only 27.6% in the control group (*p* = 0.001). When piglet age was considered in the analysis, three age groups were defined from birth to weaning: group 1 (0–7 days), group 2 (8–15 days), and group 3 (more than 15 days). Statistically significant differences in animal prevalence between diarrhea and control groups were found only in group 1, where 48.9% of the samples from piglets with diarrhea tested positive compared to 27.0% of samples from the control group (*p* = 0.019) (Fig. 2).

As for RVA, the frequency of detection of RVC was found to be significantly higher in samples from animals with diarrhea (29.2%) compared to samples from control animals (4.6%) (*p* > 0.001). When the age of the piglets was considered in the analysis, statistically significant differences (*p* = 0.001) were found between diarrhea and control groups, but only in fecal samples of group 1 (i.e. animals between 0 and 7-days-old), in which 39.0% of piglets with diarrhea tested positive compared to 10.8% in the control group (Fig. 2).

Furthermore, the mean Ct value obtained in specimens positive for RVA was significantly lower in feces from diarrheic piglets (29.9 ± 0.4) than in feces from the control group (33.5 ± 0.6) (*p* = 0.011). These differences were especially notable in animals up to 14 days of age (*p* < 0.05) (Fig. 2). Consequently, the mean Ct value for RVC in piglets with diarrhea (28.3 ± 0.5) was significantly lower (*p* = 0.002) than in the control piglets, with no scours (36.8 ± 0.3). Significant differences were only observed during the first week of lactation (28.2 ± 0.4 vs. 36.9 ± 0.1) (*p* = 0.001). Beyond 8 days of age, no RVC positive samples were found in the control group, preventing comparisons at later stages (Fig. 2).

### Frequency of detection of RV in individual pigs on farms with or without significant incidence of neonatal diarrhea

When comparing the prevalence of RVA between farms with and without high incidence of neonatal diarrhea, considering high incidence of diarrhea when more than 10–15% of the litters show scours during the lactation period, the prevalence was slightly higher in farms with diarrhea (76.1%) than in control farms (64.3%), although the difference was not statistically significant (Table 1). However, when the percentage of RVA-positive animals within each farm (i.e. within-farm positivity) was examined, significant differences were observed, with 59.2% ± 3.3% of positive samples in farms with diarrhea compared to 37.8% ± 9.1% in control farms (*p* = 0.031). On the contrary, the mean Ct values were similar in both groups and only numerical differences were observed (30.6 ± 0.6 in farms with high incidence of neonatal diarrhea vs. 33.5 ± 1.0 in control farms).

Contrary to what was observed for RVA, the prevalence of RVC was much higher in farms with neonatal diarrhea problems than in control farms (48.9% vs. 14.3%) (Table 1) and the differences were statistically significant (*p* = 0.032). In addition, the percentage of positive within-farm samples was also higher in the case farms than in the control farms (61.1% ± 4.3% vs. 40.0% ± 0.0%). However, the differences were not statistically significant, probably due to the low number of control farms (only two farms in this category were included in the study).

**Table 1:** Percentage of farms positive for RVA and RVC, and percentage of within-farm positive animals in farms with and without neonatal diarrhea problems. *Different superscripts in the same column indicate the existence of statistically significant differences*

Farm type	Number of farms	RVA		RVC	
		Positive farms (%)	Positive animals per Farm (%)	Positive farms (%)	Positive animals per Farm (%)
Case (Diarrhea)	92	76.1%	59.2% ± 3,3%^a^	48.9%^a^	61.1% ± 4.3
Control	14	64.3%	37.8% ± 9.1%^b^	14.3%^b^	40.0% ± 0.0

### Detection of RVA and RVC co-infections

**Fig. 3:**
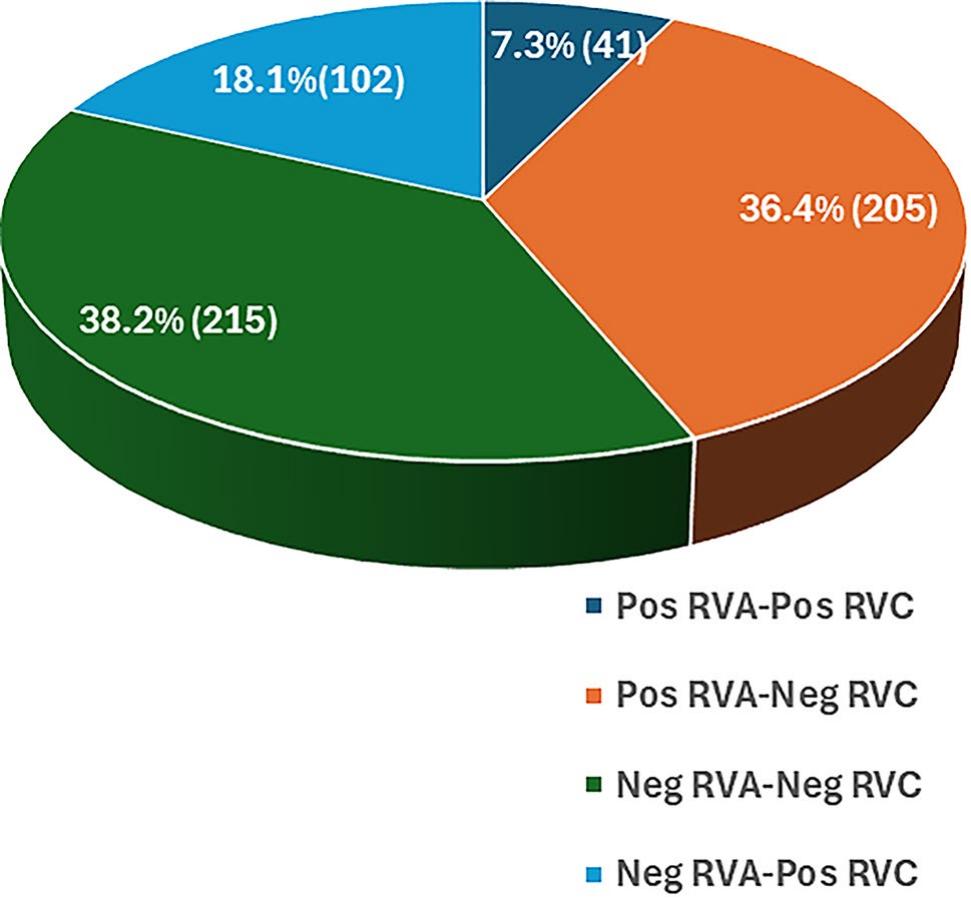
Number and percentage of fecal samples positive for either RVA or RVC, positive for both RVA and RVC, and negative for both

The frequency of detection of RVA and RVC co-infections is shown in Fig. 3. Of the 563 fecal samples that were analyzed in the study, only 41 (7.3%) tested positive for both viruses, while 54.5% were positive for one only and 38.2% were negative for both. The analysis revealed that 45.5% of the samples yielded positive or negative results for both viruses, while 54.5% were positive for one virus and negative for the other. Subsequent analysis of the data yielded a Phi coefficient of -0.177 (*p* < 0.001), indicating a negative association between the presence of the two viruses. This suggests that a positive result for one virus tends to correlate with a negative result for the other.

However, when co-infection analysis was conducted at the farm level rather than at the animal level, this trend was not observed. Thus, 31 out of the 106 farms included in the study were positive for both viruses (29.2%), while 11 farms (10.4%) were negative for both. The remaining 64 farms (60.4%) were positive for one virus and negative for the other. A subsequent statistical analysis did not reveal any statistically significant relationship between presence of both viruses (*p* = 0.113).

### Risk factors associated with RV infection

The risk factors that exerted some influence on the presence and/or RV viral load in the present study are indicated in the supplementary material (Additional file 3).

Piglet age appears to be an important risk factor for rotavirus infection (Table 2). Thus, piglets within their first week of life (0 to 7 days) had a significantly higher incidence of RVA infection than piglets older than 15 days (46.8% vs. 28.7%). The incidence of RVA infection for piglets in their second week of life did not show significant differences when compared to either of the other two groups. For RVC, piglet age was also identified as a risk factor, with younger piglets having a higher likelihood of testing positive. Specifically, the proportion of positive samples in group 1 (0 to 7 days old) was significantly higher (36.2%) than in the other two age groups (6.5% and 2.5%), and the differences were statistically significant (*p* < 0.001).

Regarding sow parity, piglets born to first-farrowing sows had a significantly higher incidence of RVA infection than those born to third or higher parity sows (51.2% vs. 39.3%) (Table 2). Conversely, the positivity of piglets born to second parity sows was not significantly different from that of piglets born to all other parity sows. When examining the influence of sow parity on the incidence of RVC infection, significant differences were found globally for the different age groups (*p* = 0.011). Specifically, piglets born to second parity sows were more likely to test positive for RVC than piglets born to older sows (32.7% vs. 18.8%). On the contrary, no significant differences were observed when comparing piglets born to gilts to those born to older sows, including second parity sows (Table 2).

**Table 2:** Influence of piglet age and Sow parity on the incidence of RVA and RVC infection. Different superscripts in the same column indicate the existence of statistically significant differences

		RVA	RVC
Piglet age (days)	0 to 7 days	46.8%^a^ (173)*	36.2%^a^ (134)
	8 to 15 days	43.0%^a, b^ (46)	6.5%^b^ (7)
	≥ 16 days	28.7%^b^ (23)	2.5%^b^ (2)
Sow parity	1st parity	51.2%^a^ (111)	28.1%^a, b^ (61)
	2nd parity	38.6%^a, b^ (39)	32.7%^b^ (33)
	> 3rd parity	39.3%^b^ (92)	18.8%^a^ (44)

* Percentage of positive samples (number of samples)

Farm size was also identified as a risk factor for the presence of RVC. Thus, larger farms (i.e., those with more than 2,000 breeding sows) were significantly more likely to test positive for RVC (81.0%) than smaller farms, where the percentage of positivity was 31.3% in those with less than 500 sows, 36.4% in those with 501 to 1,000 sows, and 37.1% in those with 1,001 to 2,000 sows (*p* = 0.003). Even more, among RVC-positive farms, those with more than 1,000 sows had a higher proportion of positive piglets than those with less than 1,000 sows (73.3% ± 4.8% vs. 37.1% ± 3.6%) (*p* = 0.011). Accordingly, these piglets showed a higher viral load in feces, as indicated by a lower mean Ct value (28.6 ± 0.8 vs. 31.7 ± 1.0) (*p* = 0.021).

The production system appeared to influence the RVA viral load in positive samples. Specifically, the mean Ct value was lower in the 34 site 1 farms, dedicated to produce weaning piglets (Ct = 29.8 ± 0.8), than in the 14 farrow-to-finish farms (Ct = 33.6 ± 1.0). These differences were statistically significant (*p* = 0.031).

In the case of RVC, the batch management production system was significantly related to the mean Ct value recorded. Thus, the 41 farms operating in weekly batches had a mean Ct value of 29.1 ± 0.7, while the 5 farms operating in a three-week batch system had a higher mean Ct value (33.9 ± 2.3).

Notably, and unlike RVA infection, RVC infection was associated with mortality rates due to enteric disease, as most farms with a lactation mortality rate below 5% over the past three and six months (70.0% and 70.4%, respectively) were RVC-negative.

Another risk factor associated with the proportion of RVC-positive animals within a farm was the downtime period between batches in the farrowing facilities. Both parameters were inversely correlated. Thus, only 50.6% ± 7.5% of the piglets in the 17 farms with a downtime of 3 or more days were positive, value significantly lower (*p* = 0.039) than the 57.7% ± 6.2% in the 17 farms with a 2-day downtime, and the 77.5% ± 7.0% in the 12 farms with only a 1-day downtime period.

On the other hand, the evaluation of the effects on RV positivity of feedback practices, aimed at ensuring that sows provide strong passive immunity to their piglets, indicated that exposure of sows to farrowing house feces influenced the incidence of RV infection. Specifically, feedback with feces from sows was associated with a higher probability of RVA detection (*p* = 0.035). In the case of RVA, all farms (*n* = 13) that conducted feedback with sow feces were positive, whereas positivity was only 71.7% in farms that did not implement this practice. Conversely, the use of feedback with placentas or dead piglets was significantly associated with a lower intra-farm RVA positivity (42.00 ± 7.94% vs. 60.19 ± 3.37%; *p* = 0.024).

Finally, it should be noted that the off-label use of a RV vaccine registered for use in ruminants did not result in any significant improvement in any of the parameters studied as indicators of RVA and RVC infection.

## Discussion

RVs, part of the *Sedoreoviridae* family, are widespread in mammals and birds and are significant causes of enteric disease in young animals. In pigs, five RV species have been described, with RVA and RVC recognized as the most pathogenic, especially in neonatal piglets [7, 13–17, 21]. While several studies have documented the prevalence and genotypes of RVA and RVC globally, most relied on clinical samples submitted to diagnostic laboratories. These often lacked key metadata, such as piglet age or sow parity, leading to potential bias and limited population-level insight.

To overcome these limitations, a systematic, year-long epidemiological study was conducted across Spain in 2023. This study incorporated geographically representative farms and accounted for the age of piglets and parity of the sows. Seasonal biases were reduced through year-round sampling. Diagnostic methods were selected based on a prior sensitivity and specificity validation study [20].

The study found a high prevalence of RVA, with 75% of farms testing positive. Regional variation showed higher prevalence in central Spain and lower in the northwest. Animal-level RVA prevalence (43.7%) was slightly lower than farm-level prevalence and aligned with previous Spanish data: 51.6% [15] and 44.9% [14], but was much higher than earlier findings, such as 26.7% [22] or 0% [17]. This increase in individual prevalence along time may reflect changes in swine management, including the use of imported hybrid genetics and higher replacement rates, which facilitate viral introduction and spread [15].

RVC was less prevalent than RVA overall. The average farm-level RVC prevalence was 44.3%, similar to that reported by Monteagudo et al. [14], though notably lower than the 71% found in Catalonia by Vidal et al. [15]. In the current study, only 28.6% of Catalonian farms tested positive, possibly due to differences in sample selection and piglet age. Animal-level prevalence for RVC was 25.4%, again lower than both farm-level RVC and RVA prevalence. Regional differences in Ct values and sample positivity were significant, paralleling findings in U.S. studies and possibly linked to regional variation in management practices [23].

When the relationship between viral presence and disease was studied, it was found that both viruses were strongly associated with diarrhea. Thus, RVC was detected in 48.9% of farms with neonatal diarrhea problems, compared to only 14.3% of control farms. At the individual level, 29.2% of the piglets with diarrhea tested positive for RVC, while only 4.3% of healthy piglets did [10, 12, 16, 24]. These results emphasize RVC’s role as a primary enteric pathogen rather than a background infection.

RVA showed similar trends. Diarrheic piglets tested positive for RVA in 48.9% of instances, compared to 27.0% in samples of piglets with normal feces. These results align with a Danish case-control study showing 25% RVA positivity in piglets with diarrhea and 6% in controls [25] and with a previous Spanish study which found 61.4% of diarrheic piglets positive versus 31.8% of healthy littermates [15]. Altogether, these results reinforce RVA’s contribution to neonatal diarrhea.

Although RVA and RVC were more common in diarrheic piglets, they were also found in healthy ones. However, mean Ct values were significantly higher in healthy animals, suggesting lower viral loads, as Ct values are inversely related to viral RNA levels. These findings suggest that viral load, rather than the mere presence of RV, plays a crucial role in disease manifestation. At this point, it is worth noting that RVA was more often detected in healthy animals than RVC. This might potentially be due to higher levels of subclinical infection or greater host immunity against RVA.

The association between viral load and disease has also been reported in human rotavirus studies, where lower Ct values (i.e., higher viral loads) correlate with more severe symptoms [26]. However, one limitation of the current study is the absence of a standard curve for converting Ct values into absolute viral copies. Standard curves—developed using known RNA concentrations—are essential for precise quantification. Without one, interpretations of viral load remain semi-quantitative.

In addition, timing of sample collection can also affect outcoming results and their interpretation. Viral shedding typically peaks 24–72 h after clinical sign onset and declines rapidly thereafter [12]. Thus, samples collected late in infection may show falsely high Ct values, underestimating actual viral loads. Accurate assessment must consider the timing of sampling in relation to clinical signs. In this line of thinking, in our study only samples from the initial phase of disease, in the first hours of diarrhea and before any treatment was implemented, were requested. However, we cannot be certain that all received samples fulfilled this requirement.

If viral load is key to disease onset, passive immunity—especially through colostrum—becomes vital. The protective role of maternal antibodies is well established in other viral infections, such as those caused by porcine coronaviruses [27, 28] and is likely to apply to RVs too. Thus, piglets lacking sufficient passive immunity acquired from colostrum may be more vulnerable to early-life infections and disease.

Indeed, the prevalence of RVC was significantly higher in one- to seven-day-old piglets than in older ones. This age-dependent pattern was consistent with findings from other European countries [29] and North America [16, 24]. Furthermore, Homwong et al. [23] reported an increased detection of RVC in piglets under three days old. This early manifestation of the disease may be due to an inadequate transfer of passive immunity from sows that are either naïve to RVC or mount a weak immune response. Further research is required to confirm this theory, but the weak RVC immunity in sows may be due to the relatively recent introduction of RVC into breeding herds, leaving many sows immunologically unprepared.

In contrast, age patterns for RVA infection differed. While RVA was slightly more common in younger piglets, the differences were not statistically significant. This contrasts with studies in North America where the prevalence of RVA increases with age and peaks in the nursery phase [10, 23]. Our findings suggest that in Spain, RVA infections often occur early, possibly due to lower maternal antibody levels or different management conditions. These early exposures may result in the development of active immunity during the suckling period [30].

One of the most intriguing findings from our data was the negative association between RVA and RVC at the individual level. That is, if a piglet tested positive for one virus, it was statistically less likely to test positive for the other. This pattern was also documented by Anderson et al., who observed that sows shed either RVA or RVC—but not both—during farrowing [31]. Similar interactions have been reported in humans, where concurrent infections with RNA and DNA enteric viruses can interfere with each other, including rotavirus vaccine strains [32]. Several mechanisms may explain this viral interference. There could be a direct competition between RVA and RVC for cellular receptors or replication resources. Alternatively, infection with one virus may trigger a strong innate immune response—such as type I and III interferon production—that creates an antiviral state in nearby cells, inhibiting secondary infections by other viral species [32].

Another objective of our study was to identify the management practices that could act as epidemiological risk factors for RV infection and disease. Risk factors are often triggering disease upon infection in production animals and their control might be critical to the success of the control programs [7]. To identify the main risk factors associated to RVA y RVC infection and disease in the swine herds in Spain we designed a questionnaire that was filled out by the farmer and the veterinarian responsible of each herd participating in the study.

Analyses of the responses obtained identified the use of feedback with feces from other sows during the gilt acclimatization protocol as a risk factor for RVA infection. All farms using this acclimatization method tested positive for RVA, compared to only 72% of farms not using it. This suggests that adult sow feces might not be a reliable source of RVA, probably because older animals might have resolved earlier infections and have lower levels of virus shedding. It is likely that those gilts, uninfected during the acclimatization period, become infected later, during pregnancy, and shed the virus more frequently around the time of farrowing [33], serving as a source of infection for their newborn piglets. However, further studies are needed to confirm this theory.

On the contrary, the use of placentas or dead piglets for feedback during gilt acclimatization showed a beneficial effect by reducing the percentage of positive samples per farm (42.00 ± 7.94% vs. 60.19 ± 3.37%; *p* = 0.024). Although RV shedding does not involve placental tissue or stillborn piglets, it is conceivable that gilt exposure to piglets that die from RV-induced diarrhea, a reliable source of RV, cause natural immunization of the gilts. However, further research is required to better understand the viral dynamics within the farm population.

In addition, the farm production system was identified as a risk factor for RVA infection, with lower mean Ct values in site 1 farms than in farrow-to-finish farms. This phenomenon has already been described in the literature [7] and could be due to the higher RVA infection pressure in farrow-to-finish farms. In these farms, breeding sows might have repeated contact with the virus, with the consequent increase in maternal immunity, which is transferred to the piglets with the colostrum, protecting them from infection and disease.

In the case of RVC, one of the main risk factors identified was the herd size. Herds with more than 2,000 breeding sows were more likely to be positive for RVC. Furthermore, when herds were divided into two categories of size (i.e., less than 1,000 breeding sows and 1,000 breeding sows or more), a greater number of positive samples per herd and a higher viral load were identified in larger farms. This finding was not unexpected, as it is more difficult for larger farms to achieve a high and homogeneous immunity level and health stability in the breeding herd [7]. A second factor associated with the presence of RVC was the mortality rate due to enteric problems in the previous six and three months. This association cannot really be considered a risk factor, but rather an indicator of the circulation of RVC in the farm and confirms its role as a primary enteric pathogen.

The last significant risk factor associated with the prevalence of RVC on the farms studied was the downtime in the farrowing rooms between batches. This is the amount of time that elapses between the cleaning and disinfection of a room and the introduction of the new group of sows into that room. As the number of days of downtime increases, the number of samples positive for RVC on a farm decreases. This inverse relationship was not surprising, as residual room contamination would be expected to be lower on farms with better cleaning and disinfection protocols and downtime between batches. Consistent with this argument, the survey data indicate that farms operating in weekly batches have higher viral load in fecal samples compared to farms operating in a three-week batch system, probably because it is easier to implement a proper cleaning and disinfection protocol and downtime period in this last type of operating system.

Finally, the use of medical prophylaxis was included in the questionnaire to evaluate its role in the prevention of RV infections. At the time this study was conducted, the only vaccine available in Spain was an inactivated vaccine originally licensed for ruminants, used in pigs with an exceptional veterinary prescription. This inactivated vaccine contains a bovine strain with the G6P1 serotype, and twenty-three of the farms participating in our study vaccinated with this inactivated vaccine. However, its efficacy in controlling RV infection in piglets appears to be limited, as no significant positive effects were found in any of the parameters evaluated for either RVA or RVC. The wide distribution of RVA and RVC in pig farms, combined with the scarce availability of immunoprophylactic tools, makes it necessary to develop specific vaccines to control the infection in pigs. For this purpose, it is desirable to have updated information not only on the prevalence but also on the circulating genotypes in order to guarantee a good protection of the pig population. Thus, future research should focus on identifying the specific RV genotypes involved in diarrhea outbreaks to better inform management and intervention strategies.

To end, it should be noted that neonatal diarrhea in pigs can be caused by a variety of enteric pathogens such as *Escherichia coli*, *Clostridium perfringens* type A and type C, *Clostridioides difficile*, and enteric coronaviruses among others [13–15]. As the objectives of this study were to determine the prevalence of RVA and RVC and to investigate the risk factors associated with the infection by both pathogens, only the presence of RVA and RVC was investigated. It cannot be excluded that other pathogens were also present in the samples analyzed. Although this circumstance can be considered an important drawback for the determination of the etiology of the diarrhea in the studied animals, it does not interfere with the main objectives of this study. It should also be mentioned that RV infection does not end with weaning, so it would be of great interest to continue studying what happens with RV infection at later stages of the pig’s life.

## Conclusions

This study highlights that RVA is a widespread pathogen in pig farms, consistently circulating among suckling piglets regardless of their age or the presence of diarrhea during lactation. The role of RVA in neonatal diarrhea appears to be associated with viral load and the proportion of infected animals. In contrast, while RVC is less prevalent in suckling piglets than RVA, its presence is strongly linked to neonatal diarrhea at very early ages. Co-infections of RVA and RVC are rare, suggesting that the presence of one virus may reduce the likelihood of detecting the other. Several key factors influence the presence of these viruses, including piglet age, sow parity, farm production system, farm size, and the number of downtime days in farrowing rooms, which is particularly relevant for RVC infection. To enhance disease prevention and control, up-to-date data on prevalence and circulating genotypes are crucial.

## Supplementary Information

Below is the link to the electronic supplementary material.

Supplementary Material 1

Supplementary Material 2

Supplementary Material 3

## Data Availability

The datasets used and/or analysed during the current study are available from the corresponding author on reasonable request.

## Abbreviations

RVs: Rotaviruses
RVA: Rotavirus A
RVC: Rotavirus C
VPs: viral structural proteins
NSPs: nonstructural proteins
PBS: Phosphate Buffer Saline
HBGAs: histo-blood group antigens
Ct: Cycle threshold
