# Supplementary material for: Prevalence and impact of Rotavirus A and C in suckling piglets from Spanish farms: an epidemiological study
Source: Porcine Health Manag. 2025 Oct 30;11:54. doi: 10.1186/s40813-025-00468-z (PMC12573817; doi:10.1186/s40813-025-00468-z)
Supplement: Supplementary file 1 — Supplementary Material 1 [file 40813_2025_468_MOESM1_ESM.docx]

**Additional file 1:** National swine inventory distribution according to the number of breeding sows per Autonomous Community (region), and the number of sampled farms.

| **Autonomous Community (Region)** | **Number of breeding sows** | **Percentage of total inventory** | **Objective number of farms to be sampled** | **Actual number of farms sampled by region**  **(diarrhea + control)** |
| --- | --- | --- | --- | --- |
| Aragon | 636.280 | 23,8% | 23 | 23 (22+1) |
| Catalonia | 595.857 | 22,3% | 22 | 21 (18+3) |
| Castile and Leon | 465.068 | 17,4% | 17 | 16 (14+2) |
| Andalusia | 245.435 | 9,2% | 10 | 10 (8+2) |
| Castile-La Mancha | 163.650 | 6,1% | 7 | 7 (5+2) |
| Murcia | 152.086 | 5,7% | 6 | 6 (6+0) |
| Extremadura | 150.989 | 5,6% | 6 | 8 (7+1) |
| Galicia | 88.353 | 3,3% | 5 | 7 (6+1) |
| Valencian Community | 77.216 | 2,9% | 2 | 4 (3+1) |
| Navarre | 71.015 | 2,7% | 2 | 4 (3+1) |
| Others | 27.460 | 1,0% | 0 | 0 |
|  | **2.673.409** | **100,0%** | **100** | **106 (92+14)** |

**Source:** MINISTRY OF AGRICULTURE, FISHERIES AND FOOD. General Directorate of Agricultural Productions and Markets. Data as inventory in December 2022.
