# Supplementary material for: Prevalence and impact of Rotavirus A and C in suckling piglets from Spanish farms: an epidemiological study
Source: Porcine Health Manag. 2025 Oct 30;11:54. doi: 10.1186/s40813-025-00468-z (PMC12573817; doi:10.1186/s40813-025-00468-z)
Supplement: Supplementary file 2 — Supplementary Material 2 [file 40813_2025_468_MOESM2_ESM.docx]

**Additional file 2. Variables used to assess the risk factors of rotavirosis in lactating piglets.**

| **Variables** | **Definition** | **Category** |
| --- | --- | --- |
| Farm size | Number of productive sows | Less than 500 sows  501 to 1,000 sows  1,001 to 2,000 sows  More than 2,000 sows |
| Productive orientation of the farm | Kind of animals produced by the farm | Piglets at weaning  Growers to 20-30kg  Fatteners |
| Batch production system | Frequency in weeks of the farrowing batches | Weekly batch  3-week batch  4-week batch  others |
| Replacement rate | Percentage of sows replaced by gilts in the last year | Less than 25%  26% to 33%  34% to 49%  50% or more |
| Is neonatal diarrhea an issue in your farm? | If you have more than 10-15% of litters with diarrhea during lactation period | Yes  No |
| Average pre-weaning mortality % during the last 6 months | Mortality % of suckling piglets | Less than 5%  6% to 10%  11% to 15%  16% or more |
| Average pre-weaning mortality % during the last 3 months | Mortality % of suckling piglets | Less than 5%  6% to 10%  11% to 15%  16% or more |
| Average pre-weaning mortality % due to enteric disorders during the last 6 months | Mortality % of suckling piglets due to diarrhea | Less than 5%  6% to 10%  11% to 15%  16% or more |
| Average pre-weaning mortality % due to enteric disorders during the last 3 months | Mortality % of suckling piglets due to diarrhea | Less than 5%  6% to 10%  11% to 15%  16% or more |
| Sow vaccination scheme | Associated with diseases related to enteric pathology (MULTIPLE CHOICE) | Colibacilosis  *Clostridium perfringens* type C  *Clostridium perfringens* type A  *Clostridioides difficile*  Rotavirus  Sows not vaccinated against enteric pathogens |
| Piglet vaccination scheme during lactation | Associated with diseases related to enteric pathology (MULTIPLE CHOICE) | Colibacilosis  Rotavirus  Others  Piglets not vaccinated against enteric pathogens |
| Days of downtime period between batches in the farrowing room | It is the time that elapses between the end of the disinfection of the room and the entry of a new batch of sows to farrow | 0 days  1 day  2 days  3 or more days |
| Cross-fostering policy with piglets after farrowing | Check the option that best suits your driving. | Efforts are made to ensure all the piglets take colostrum from their own mother, and then, minimum number of piglets are moved.  It is ensured that all the piglets take colostrum from their own mother, after which, a lot of piglets are moved just to equal piglets per litter.  It is ensured that all the piglets take colostrum from their own mother, after which, a lot of piglets are moved to equal the litters with piglets of similar size.  Efforts are made to ensure that all the piglets take colostrum from their own mother, but high hyper- prolific sows force me to perform extensive cross-fostering, moving complete litters forward.  It is not essential that the piglets take colostrum from their own mother, but piglets are moved as soon as possible to equal the litters with piglets of similar size and to guarantee access to the teat. |
| Type of management carried out on the farm to acclimate gilts before entering the breeding unit | Adaptation method used to acclimate gilts to the pathogens present on the breeding sows (MULTIPLE CHOICE) | Feed-back with feces of piglets with diarrhea.  Feed-back with feces from other sows.  Feed-back with placentas or dead piglets.  Feedback with culling sows due to slaughter.  Adaptation only with vaccines. |
