# Supplementary material for: Prevalence and impact of Rotavirus A and C in suckling piglets from Spanish farms: an epidemiological study
Source: Porcine Health Manag. 2025 Oct 30;11:54. doi: 10.1186/s40813-025-00468-z (PMC12573817; doi:10.1186/s40813-025-00468-z)
Supplement: Supplementary file 3 — Supplementary Material 3 [file 40813_2025_468_MOESM3_ESM.docx]

**Additional file 3:** Risk factors associated with the prevalence of RVA and RVC infection or viral load found. Different superscripts in the same column and for the same evaluated parameter denote significant differences

| **Parameter** | **Description of the evaluated parameter (possible categories)** | **Results to RVA** | | | **Results to RVC** | | |
| --- | --- | --- | --- | --- | --- | --- | --- |
|  |  | **Farm positivity** | **Intra-farm positivity percentage** | **Average Ct value of positive samples** | **Farm positivity** | **Intra-farm positivity percentage** | **Average Ct value of positive samples** |
| Farm size | Less than 500 sows | n.s. | n.s. | n.s. | 31.3%^a^ | 37.1% ± 3.6%^a^ | 31.7 ± 1.0^a^ |
|  | 501 – 1,000 | n.s. | n.s. | n.s. | 36.4%^a^ |  |  |
|  | 1,001 – 2,000 | n.s. | n.s. | n.s. | 37.1%^a^ | 73.3% ± 4.8%^b^ | 28.6 ± 0.8^b^ |
|  | More than 2,000 sows | n.s. | n.s. | n.s. | 81.0%^b^ |  |  |
| Productive orientation of the farm | Piglets at weaning | n.s. | n.s. | 29.8 ± 0.8ª | n.s. | n.s. | n.s. |
|  | Piglets to 20 kg | n.s. | n.s. | 30.9 ± 0,9ª^,b^ | n.s. | n.s. | n.s. |
|  | Fatteners | n.s. | n.s. | 33.6 ± 1.0^b^ | n.s. | n.s. | n.s. |
| Batch production system | 1-week batch system | n.s. | n.s. | n.s. | n.s. | n.s. | 29.1 ± 0.7^a^ |
|  | 2-week batch system | n.s. | n.s. | n.s. | n.s. | n.s. | n.i. |
|  | 3-week batch system | n.s. | n.s. | n.s. | n.s. | n.s. | 33.9 ± 2.3^b^ |
| Diarrhoea as an issue during lactation period | Yes, diarrheic sample | n.s. | 59.2% ± 3.3%^a^ | n.s. | 49.5%^a^ | n.s. | n.s. |
|  | No, normal faeces | n.s. | 37.8% ± 9.1%^b^ | n.s. | 14.3%^b^ | n.s. | n.s. |
| Mortality due to enteric disease over last 3 months | Less than 5% | n.s. | n.s. | n.s. | 30.0%^a^ | n.s. | n.s. |
|  | 6% – 10% | n.s. | n.s. | n.s. | 64.4%^b^ | n.s. | n.s. |
|  | 11% - 15% | n.s. | n.s. | n.s. |  | n.s. | n.s. |
|  | ≥ 16% | n.s. | n.s. | n.s. |  | n.s. | n.s. |
| Mortality due to enteric disease over last 6 months | Less than 5% | n.s. | n.s. | n.s. | 29.6%^a^ | n.s. | n.s. |
|  | 6% – 10% | n.s. | n.s. | n.s. | 60.8%^b^ | n.s. | n.s. |
|  | 11% - 15% | n.s. | n.s. | n.s. |  | n.s. | n.s. |
|  | ≥ 16% | n.s. | n.s. | n.s. |  | n.s. | n.s. |
| Downtime period between batches in the farrowing room | 0 days, no downtime period | n.s. | n.s. | n.s. | n.s. | n.i. | n.s. |
|  | 1 day | n.s. | n.s. | n.s. | n.s. | 77.5% ± 7.0%^a^ | n.s. |
|  | 2 days | n.s. | n.s. | n.s. | n.s. | 57.7% ± 6.2%^a^ | n.s. |
|  | 3 or more days | n.s. | n.s. | n.s. | n.s. | 50.6% ± 7.5%^b^ | n.s. |
| Feed-back with faeces from sows | Yes | 100%^a^ | n.s. | n.s. | n.s. | n.s. | n.s. |
|  | No | 71.7%^b^ | n.s. | n.s. | n.s. | n.s. | n.s. |
| Feed-back with placentas or dead piglets | Yes | n.s. | 42.0% ± 7.9%^a^ | n.s. | n.s. | n.s. | n.s. |
|  | No | n.s. | 60.2% ± 3.4%^b^ | n.s. | n.s. | n.s. | n.s. |

n.s.: Not significant (p≥0.05). n.i..: Not investigated.:
